# Supplementary material for: PASA: Proteomic analysis of serum antibodies web server
Source: PLoS Comput Biol. 2021 Jan 25;17(1):e1008607. doi: 10.1371/journal.pcbi.1008607 (PMC7861515; doi:10.1371/journal.pcbi.1008607)
Supplement: Attachment — Submitted filename: PASA_V7_point_by_point_letter.docx [file pcbi.1008607.s001.docx]

SHMUNIS SCHOOL OF BIOMEDICINE AND CANCER RESEARCH

Dear Dr. Mihaela Pertea,

Enclosed please find our revised manuscript entitled “**PASA: Proteomic Analysis of Serum Antibodies web server**” (PCOMPBIOL-D-20-01304) by Oren Avram, Aya Kigel, Anna Vaisman-Mentesh, Sharon Kligsberg, Shai Rosenstein, Yael Dror, Tal Pupko, and Yariv Wine.

We would like to begin by thanking you and the reviewers for the helpful, productive, and constructive feedback on our manuscript and for the many insightful comments that helped us to further improve our study and the clarity of the text. We revised the manuscript and incorporated all comments and suggestions.

Our detailed point-by-point reply for all comments is given below. The comments of the reviewers are in bold, our answers are detailed below each comment followed by the text (when available) copied from the revised manuscript, in *italics*.

We hope you find our revised manuscript appropriate for publication in *PLOS Computational Biology*. We thank you in advance for your consideration and look forward to your response.

**Sincerely,**

**Dr. Yariv Wine on behalf of all authors**

# Referee #1 (Remarks for Author):

**Summary: In this manuscript, Avram and Kigel and colleagues, report the implementation of the PASA web server that is stated to provide a robust computational platform for the analysis and integration of data obtained from proteomics of serum antibodies. Specifically, PASA enables the mapping of peptides derived from antibodies raised against a specific antigen to corresponding antibody sequences. Can the authors identify the maxquant version used and the specific maxquant parameters. Furthermore the maxquant parameters files should be provided in the output so that users may reproduce their findings in the future.**

We thank the reviewer for highlighting this important point. The current version of MaxQuant used by PASA (1.6.14) is now mentioned in the web server page (rather than in the manuscript itself, in case we would like to update it in the future). In addition, we added the MaxQuant configuration file (mqpar.xml) of both the elution and flow-through to the output text folder together with all other textual results generated by PASA, see “Download results (both textual & visual)”.

**Furthermore, the versatility of the webserver remains opaque to the reviewer. Can the webserver also be used if one does not have antigen-specific data? So, just a VDJ-database and MS/MS raw files?**

We thank the reviewer for this comment. The suggestion to use PASA on antigen-specific data stems from the experimental complexity of the sample. The polyclonal antibody population in the serum is highly complex and represents antibodies that target many antigens as well as “housekeeping” antibodies that may exhibit poly-reactive properties. Enriching the sample for antigen-specific antibodies help reduce this complexity and narrow down the search space to focus only on the antibodies of interest in the chosen model. Thus, we do not recommend applying PASA on antibodies that are not antigen-specific. Nevertheless, following the reviewer’s comment, we acknowledge the applicable potential for the analysis of non-antigen-specific samples. Thus, we implemented in the revised version a workaround that allows the integration of BCR-seq data and proteomic data derived from samples that are not antigen-specific (given that the input includes the required V gene sequence database and a peptides list, rather than MS/MS raw files). To this end, the user will need to run MaxQuant as stand-alone (not via PASA) and submit to PASA the output peptides list as the “elution peptides list”, together with an empty “flow-through peptide list”. This additional feature will leverage PASA capabilities to analyze and integrate BCR-Seq and MS/MS data to facilitate the identification of serum antibodies. The utilization of this new capability is now detailed step by step in the “Non antigen-specific analysis” section at the Overview page of the web server.

**Can the authors provide runtime analyses for common file sizes?**

We thank the reviewer for this comment. We added a “Running time” section to the overview page and we also added an appropriate description to the manuscript (page 4, line 79-85 in the revised manuscript):

*“PASA’s running time may vary as it depends on the number of replicates and databases used. For instance, the analysis of the example input files (elution and flow-through triplicates, each triplicate of size ~6GB, together with three V gene sequence database, each of size 35 MB) took less than three hrs. If a user runs MaxQuant as stand-alone (i.e., not via PASA) and submits to PASA the output peptides list (instead of the raw mass spectrometry data files), the running time is dramatically reduced to no more than a few minutes (using the example V gene sequence database).”*

**Why is there no command line version which would enable parallelization etc?**

We thank the reviewer for this comment. We have originally provided the option to download PASA pipeline scripts from <https://github.com/orenavram/PASA>. Following the reviewer’s comment, we have further clarified how to run the pipeline locally on the repository’s main page. Of note, the pipeline is being fully parallelized whenever possible: the elution and flow-through files are being processed simultaneously, each of them is being parallelized on 20 different threads. In the “Availability and Future Directions” section of the revised manuscript we now write (page 5, line 110-112 in the revised manuscript):

*“PASA is freely available for noncommercial users as a web server at* [*https://pasa.tau.ac.il*](https://pasa.tau.ac.il) *and as a command line application as well. The source code of PASA is written in Python and is available at* [*https://github.com/orenavram/PASA*](https://github.com/orenavram/PASA)*.”*

Please note that this part was moved from the abstract to the last section as required by the journal format.

**What is the novelty of the PASA tool? Have you gained any biological insights using PASA?**

The PASA tool is a unique computational platform that enables non-expert users to upload VDJ sequencing and MS/MS data directly to the webserver that processes, interprets and integrates sequencing and mass-spec data in a user-friendly manner. To the best of our knowledge, there is no available computational platform similar to PASA. Given the increasing interest in the proteomic analysis of serum antibodies we established this platform that will propel the research in the community and enable non-computational research groups to generate data that otherwise were reluctant to generate. Proteomic analysis of serum (and other bodily fluids) antibodies were shown to provide invaluable insights regarding the nature of the immune response. In the previous version of the manuscript, in the Discussion section we mentioned several pivotal examples. To clarify the important biological insights that can be generated using Ig-seq, we added a pivotal example to the revised Introduction section as well and we now write (page 3, line 55-57 in the revised manuscript):

*“Since its establishment [2], Ig-Seq has opened new research avenues that yielded invaluable insights regarding the development of the immune response during disease and following vaccination [12-17].”*

However, all of these studies analyzed and integrated the data using low throughput methods (excel or other possible software), which lack data integration capabilities, not to mention scalability. Such data analysis required the user to be an expert in computational analysis thus, preventing many research groups to utilize this methodology. What PASA does is to provide a user-friendly computational platform that makes the data processing and integration very easy to be used by non-expert users. Moreover, our previous developed tool called ASAP (<https://asap.tau.ac.il/>) provides an additional platform enabling the users to easily generate the VDJ data required for the MS/MS analysis ([Avram, *et al*, 2018](https://doi.org/10.3389/fimmu.2018.01686)).

**Line 45: please provide original references for the statement that the CDR3H contributes most to antigen binding (for example Xu *et al*., 2000, Immunity (last author: Mark Davis)).
Line 46: Please provide a reference for the statement: CDRH3 is often used as a unique identifier to determine antibody clonality**

We have added the references as suggested by the reviewer for line 45 (line 43 in the revised manuscript) and 10.1186/s12865-014-0040-5, 10.1371/journal.pcbi.1002388 for line 46 (line 44 in the revised manuscript).

[**https://pasa.tau.ac.il/gallery.html**](https://pasa.tau.ac.il/gallery.html)**: color scheme for the Isotype Distribution pie chart is not color blind friendly**

This is a very import point and we apologize for not accommodating the needs of color blinded users. We have revised the color scheme accordingly (precisely, we now use the “colorblind” from <https://seaborn.pydata.org/tutorial/color_palettes.html>).

[**https://pasa.tau.ac.il/gallery.html:**](https://pasa.tau.ac.il/gallery.html:) **the y-axis “% of total” Percent of total what? All the peptides or ig-related peptides?**

Fixed. We have revised the y-axis title to “Relative frequency (in %)”. In other words, the total sum of all the bars in the x axis is 100 (%) and for each column, we provide what is its contribution for the total.

[**https://pasa.tau.ac.il/index.html**](https://pasa.tau.ac.il/index.html) **BCR-Seq DB tooltip: link to the ASAP format goes to the same (index.html) page**

Thank you for pointing out this problem. We have fixed the link to point the ASAP web page ([**https://asap.tau.ac.il/index.html**](https://asap.tau.ac.il/index.html)).

[**https://pasa.tau.ac.il/index.html**](https://pasa.tau.ac.il/index.html)**: “Please notice that since the raw files are (very) heavy.” — English?**

Fixed (changed to “large”).

**Line 92: “Proteomics intensity with respect to each BCR-seq database intensity” — what does BCR-seq database intensity mean?**

Indeed, the description given in the original manuscript was unclear. Essentially, BCR-seq database intensity, refers to the relative abundance of the antibody clone in the BCR-Seq data. We clarified this point in the manuscript, and in the revised version we now write (page 5, line 104-105 in the revised manuscript):

*“Proteomics intensity with respect to each antibody sequence frequency as observed in the BCR-seq database”*

# Referee #2 (Remarks for Author):

**Wine *et al*. present PASA (proteomic analysis of serum antibodies) a web server for proteomic antibody repertoire analysis. Presumably the server was designed for human samples -no information is provided on whether it might work for antibody repertoires from other species.**

Thank you for pointing out this – we have revised the manuscript and mentioned that PASA is good for human and murine data (page 3, line 62-64 in the revised manuscript):

*“Here, we developed PASA, an integrative approach that combines NGS with serum-antibody proteomics (derived from either human or murine), that allows mapping, analysis, and integration of peptides data with reference to individual specific BCR-Seq data.”*

**PASA is very user friendly and enables to process and assign proteomic data using a specimen specific V gene data base. This is a tool that is likely to be very useful for the community and publication is recommended. I wish the V gene DNA sequence was more versatile (only data in ASAP is accepted) but that is a minor complaints. The manuscript is very well written and to the point.**

We thank the reviewer for the positive feedback. We hope that in the future, uniform established formats for such data become standardized, and then, PASA will accommodate these formats.
